# Toxicity of Pesticides Applied in European Vineyards on *Anagyrus vladimiri* and *Trichogramma evanescens*, Parasitoids of *Planococcus ficus* and *Lobesia botrana*

**DOI:** 10.3390/insects14120907

**Published:** 2023-11-24

**Authors:** Ramzi Mansour, Anna Lena Bauer, Maryam Goodarzi, Christoph Hoffmann

**Affiliations:** 1 Julius Kühn-Institute—Federal Research Institute for Cultivated Plants, Institute for Plant Protection in Fruit Crops and Viticulture, Geilweilerhof, 76833 Siebeldingen, Germany; 2 Higher Institute for Preparatory Studies in Biology-Geology (ISEP-BG), Section of Biological Sciences, University of Carthage, Tunis, La Soukra 2036, Tunisia

**Keywords:** vine mealybug, European grapevine moth, biological control, pesticide toxicity, vineyard integrated pest management

## Abstract

**Simple Summary:**

The toxicity of four insecticides and one fungicide commonly applied in German (European) vineyards was evaluated on pupae and adults of the parasitoids *Anagyrus vladimiri* and *Trichogramma evanescens*. The insecticides lambda-cyhalothrin, flupyradifurone, acetamiprid, and cyantraniliprole and the fungicide spiroxamine did not significantly affect the development of the pupal stage inside mealybug mummies or the emergence of the parasitoid *A. vladimiri.* The highest mortality percentages of emerged *A. vladimiri* parasitoids were induced by flupyradifurone, acetamiprid, or spiroxamine. All pesticides, except cyantraniliprole, significantly affected the development of the pupal stage and the emergence of the parasitoid *T. evanescens*. Regarding direct contact toxicity, the highest percentages (100%) of *A. vladimiri* adult parasitoid mortality were obtained in the flupyradifurone, acetamiprid, and spiroxamine treatments (all three classified as harmful), while the lowest mortality percentages were observed in lambda-cyhalothrin and cyantraniliprole (both classified as slightly harmful). The highest percentages (100%) of adult *T. evanescens* mortality were found in the flupyradifurone, acetamiprid, and spiroxamine treatments, while the lowest mortality percentages were observed in lambda-cyhalothrin and cyantraniliprole treatments.

**Abstract:**

Risk assessments of chemical pesticides toward natural enemies are crucial for ensuring sustainable grapevine-integrated pest management. In this context, laboratory experiments were conducted to evaluate the toxicity of four insecticides (lambda-cyhalothrin, flupyradifurone, acetamiprid, and cyantraniliprole) and one fungicide (spiroxamine) commonly applied in German (European) vineyards on the pupae and adults of both *Anagyrus vladimiri*, a parasitoid of the vine mealybug *Planococcus ficus*, and *Trichogramma evanescens*, a parasitoid of the European grapevine moth, *Lobesia botrana*. The tested pesticides did not significantly affect the development of the pupal stage inside mealybug mummies or the emergence of the parasitoid *A. vladimiri*. The pesticides flupyradifurone, acetamiprid, and spiroxamine resulted in the highest mortality percentages for all emerged *A. vladimiri* parasitoids at 8 and 10 days after treatment compared with either in lambda-cyhalothrin or cyantraniliprole. However, all pesticides, except the diamide insecticide cyantraniliprole, significantly affected the development of the pupal stage and the emergence of the parasitoid *T. evanescens*. The percentages of *T. evanescens* emergence following the application of the fungicide spiroxamine or either lambda-cyhalothrin or flupyradifurone were significantly higher than those observed in the acetamiprid treatment. Regarding direct contact toxicity, the highest percentages (100%) of *A. vladimiri* adult parasitoid mortality were obtained in the flupyradifurone, acetamiprid, and spiroxamine treatments, while the lowest mortality percentages were observed in lambda-cyhalothrin, cyantraniliprole, and untreated control treatments. According to the IOBC classes of toxicity, flupyradifurone, acetamiprid, and spiroxamine were classified as harmful, while both lambda-cyhalothrin and cyantraniliprole were classified as slightly harmful to *A. vladimiri* adults. As such, all pesticides had a significant impact on the survival of exposed *T. evanescens* adults. The highest percentages of adult *T. evanescens* mortality were obtained in the flupyradifurone, acetamiprid, and spiroxamine treatments, with the fungicide spiroxamine resulting in significantly higher mortality percentages than either flupyradifurone or acetamiprid, while the lowest mortality percentages were found in the lambda-cyhalothrin and cyantraniliprole treatments. Therefore, applying the insecticides acetamiprid and/or flupyradifurone and the fungicide spiroxamine should be avoided when *A. vladimiri* and/or *T. evanescens* are naturally present or released in grapes. The insights gained from these two easy-to-rear parasitoid species allow analogous conclusions to be drawn for closely related species in vineyards belonging to either family Encyrtidae or Trichogrammatidae, which are not easy to rear. Interestingly, using the safer insecticides lambda-cyhalothrin and/or cyantraniliprole could be compatible with both parasitoid species, which could be sustainably exploited in either conservation or augmentative biological control in vineyards.

## 1. Introduction

Grapevine (*Vitis vinifera* L.) production has always been affected by a number of arthropod pests, including insects and mites, and pathogenic microorganisms such as fungi, bacteria, oomycetes, and viruses, resulting in severe crop damage and substantial economic losses [1,2,3,4]. For decades, both insect species, the vine mealybug *Planococcus ficus* (Signoret) (Hemiptera: Pseudococcidae) and the European grapevine moth *Lobesia botrana* (Denis and Schiffermüller) (Lepidoptera: Tortricidae), have been considered among the most damaging, economically important arthropod pests of grapevines worldwide [5,6,7,8].

As a plant phloem feeder, *P. ficus* excretes honeydew that supports the growth of sooty mold fungus, which negatively impacts the quality of grape clusters, while its feeding on leaves can result in defoliation [3,5]. Furthermore, vine mealybugs have been shown to act as potential vectors of grapevine viruses, mainly including Leafroll Associated Viruses (GLRaVs) that cause grapevine leafroll disease (GLD) [9,10,11]. Similar to *P. ficus*, attacks on grapevines by *L. botrana* populations have been proven to induce severe damage in immature, ripening, and ripe berries with major economic losses, especially in European vineyards [3,12,13]. This is mainly due to the carpophagous (fruit-feeding) generations, which are the most destructive compared with the first (flower-feeding) generation, with additional indirect damage caused by a resulting infection by the gray mold fungus *Botrytis cinerea* [13,14].

These serious grape health issues have driven scientists to conduct relevant research studies to find out the most effective, eco-friendly, and sustainable pest management options against both the vine mealybug and the European grapevine moth. Chemical control through pesticide treatments has been among the most applied options to cope with both *P. ficus* and *L. botrana* infestations in vineyards worldwide [1,5,6,12]. Nevertheless, chemical pesticide treatments cannot always be regarded as a successful approach because of two major constraints that can compromise their use: the resistance of the target insect pest to a number of active substances and adverse side effects in non-target beneficial arthropods, as well as other ecosystem components [1,6,15,16]. For these reasons, there has been increasing evidence that chemical pesticide treatments against both pest species should be replaced with other environmentally safe, sustainable control approaches in vineyards [6,8,17,18].

In this context, biological control using species-specific parasitoids and/or predators has always been considered a very promising eco-friendly option to effectively control *P. ficus* and *L. botrana* in vineyards. More specifically, *Anagyrus vladimiri* Triapitsyn (formerly known as *Anagyrus* (sp. near) *pseudococci* (Girault)) (Hymenoptera: Encyrtidae) is considered among the most common and effective parasitoids of vine mealybugs in vineyards [5,15,18,19]. As such, *Trichogramma* sp. (Hymenoptera: Trichogrammatidae) egg parasitoids, which have long been used against a wide range of lepidopteran pests [20], are considered suitable biocontrol agents to be mass-reared for releases against *L. botrana* in European vineyards [8]. Native *Trichogramma* species that have been found to be associated with grapevine moths in European (German and/or French) vineyards include, among others, *Trichogramma evanescens* Westwood, *T. cacoeciae* Marchal, *T. daumalae* Dugast and Voegelé, *T. principium* Sugonjaev and Sorokina, *T. brassicae* Bezdenko, and *T. pintoi* Voegelé [21,22]. For example, *T. evanescens*, released at 800 points per hectare, parasitized more *L. botrana* eggs of the first generation than *T. cacoeciae* and significantly reduced damage to grapes in French vineyards, while *T. cacoeciae* was more efficient than *T. evanescens* in the second generation of this pest [23]. However, it is worth mentioning that evaluating the compatibility (selectivity) of pesticides with natural enemies in a target insect pest should always be considered when developing and implementing integrated pest management (IPM) programs, including in vineyards [6,8,24,25].

From this perspective, the present study was performed and aimed at evaluating the toxicity of four insecticides and one fungicide commonly applied in German (European) vineyards to the pupae and adults of both (i) *A. vladimiri*, a parasitoid of the vine mealybug, and (ii) *T. evanescens*, a parasitoid of the European grapevine moth, to enhance sustainable integrated pest management programs for both insect pests in vineyards. Both parasitoid species also might serve as model organisms to draw analogous conclusions regarding sister species from the same genus that cannot be reared commercially, e.g., *A. schönherri* (Westwood), a parasitoid of the apple mealybug *Phenacoccus aceris* (Signoret) [26], which is an important vector of grapevine leafroll disease in Europe [27].

## 2. Materials and Methods

### 2.1. Insects and Chemical Pesticides

Insect individuals of the encyrtid solitary koinobiont parasitoid *A. vladimiri* (Koppert B.V., Berkel en Rodenrijs, the Netherlands) were obtained as pupae (parasitized mealybugs), about 6–7 days before eventual parasitoid emergence. *Trichogramma evanescens* parasitoids (Sautter & Stepper GmbH, Ammerbuch, Germany) were also provided as pupae (parasitized eggs of the Angoumois grain moth *Sitotroga cerealella* (Olivier) (Lepidoptera: Gelechiidae) glued on paper cards) about 3 days before adult emergence. All parasitoid pupae were stored under controlled laboratory conditions (25 ± 1 °C; 60% relative humidity; 12 h:12 h L:D photoperiod) until the experiments were initiated.

Five pesticides (four insecticides and one fungicide (Table 1)) commonly used in European vineyards against a number of major insect pests and/or fungal pathogens were applied at their field-recommended rates to be assessed for their toxicity on the pupal and adult stages of *A. vladimiri*, a common solitary endoparasitoid of the vine mealybug *P. ficus*, and *T. evanescens*, a common egg endoparasitoid of the European grapevine moth *L. botrana*.

### 2.2. Pesticide Toxicity toward Anagyrus vladimiri

For the parasitoid pupal or adult stages experiments, glass Petri dishes (14 cm width; internal surface area of 396.8 cm^2^) were prepared by taping a 1 cm strip of double-sided adhesive tape to the middle of the bottom plate.

Eight mummified mealybugs, each containing one pupa of the parasitoid *A. vladimiri*, were placed onto the tape of each Petri dish. Sea sand was added to prevent the emerging parasitoids from sticking to the tape. Then, the lower part of the plate was sprayed with each of the five pesticide active substances mentioned previously, using the Tray Spray Cabinet (Schachtner Fahrzeug-und Gerätetechnik, Ludwigsburg, Germany) (sprayed area: 153.9 cm^2^). A singular nozzle TeeJet^®^ TP80 (TeeJet^®^ Technologies, Schorndorf, Germany) was used to perform 1-dimensional vertical spraying on top of the Petri dishes. The speed was 2.75 km/h, with a pressure of 2.5 bars. Each pesticide was applied at its field-recommended dose in German vineyards. An untreated control was sprayed with tap water. Afterward, a 90% honey solution Bio Waldhonig (Alnatura, Darmstadt, Germany) was prepared and distributed in minute droplets on a 90 mm surface of the Petri dish that was placed over the mummies within the 140 mm Petri dish (inner surface area: 183.8 cm^2^ treated 63.6 cm^2^). During the whole period of the assessments, the plates were stored in a climate chamber (25 ± 1 °C; 60% relative humidity; 12 h:12 h L:D photoperiod). Six replicates were performed per treatment, with a total of 288 pupae used for all six treatments. The total number of emerged and dead parasitoid individuals per replicate was counted visually using a binocular microscope ZEISS Stemi 508 (ZEISS Gruppe, Oberkochen, Germany) every 24 h for a period of 14 days after treatments.

Experiments to assess contact toxicity on adult parasitoids were performed following the standard principles adopted by the IOBC/WPRS working group, “Pesticides and Beneficial Organisms” [30,31]. The five aforementioned pesticides and tap water (untreated control) were sprayed following the same procedure adopted for the previously described experiments on the pupae using the Tray Spray Cabinet (Schachtner Fahrzeug-und Gerätetechnik, Ludwigsburg, Germany). As soon as the sprays dried, a 90% honey solution was distributed on the upper surface of each Petri dish, within which, four 1-day-old *A. vladimiri* males and four 1-day-old *A. vladimiri* females were introduced to be exposed to the fresh dry pesticide/tap water surface. The Petri dishes with parasitoids were held in a climate chamber under controlled conditions (25 ± 1 °C; 60% relative humidity; 12 h:12 h L:D photoperiod). Five replicates were performed per each of the six treatments, with a total of 240 adult parasitoids used in the experiment. Mortality of adult parasitoids was evaluated at 24, 48, and 72 h after treatment using a binocular microscope.

Based on their respective mortality %, all pesticides were classified into four categories corresponding to the IOBC classes of toxicity: category 1: harmless (<30% mortality); category 2: slightly harmful (30–79% mortality); category 3: moderately harmful (80–99% mortality); and category 4: harmful (>99% mortality) [30,31].

### 2.3. Pesticide Toxicity toward Trichogramma evanescens

Pesticide toxicity toward the pupal stage of the parasitoid *T. evanescens* was assessed following the same pesticide/tap-water-spraying procedure used for evaluating toxicity on *A. vladimiri* pupae, as described above. However, after pupae were placed on an internal surface of a glass Petri dish plate and sprayed using the Tray Spray Cabinet, each small paper card containing 10 treated pupae was transferred to a clean glass tube (height: 8.0 cm; diameter: 2.0 cm; internal surface area: 53.38 cm^2^) and left under controlled laboratory conditions (25 ± 1 °C; 70 ± 10% relative humidity; and 14 h:10 h (L:D) photoperiod). Six replicates were performed per treatment, with a total of 60 *T. evanescens* pupae considered for each treatment. The total number of emerged parasitoid individuals per replicate was counted visually using a binocular microscope twice a day for a period of 7 days after treatment.

However, pesticide contact toxicity toward 1-day-old *T. evanescens* adults was evaluated using a dry film residue method, as described by Desneux et al. [32] and Wang et al. [33]. In total, 500 μL of acetone solution of each pesticide was deposited in the internal surface of each glass tube (height: 8.0 cm; diameter: 2.0 cm; internal surface area: 53.38 cm^2^), while pure acetone was introduced to each untreated control tube, which was rotated to avoid the presence of droplets on its wall. All tubes were stored for 1 h under the aforementioned controlled conditions to allow for the evaporation of acetone. Then, 10 *T. evanescens* adults (without considering their sex) were introduced to each tube, and a 90% honey solution was added to the plastic strip, which allowed for air circulation in the tube. All tubes were left in controlled laboratory conditions (25 ± 1 °C; 70 ± 10% relative humidity; and 14 h:10 h (L:D) photoperiod). Seven treatments (five types of acetone pesticide solutions, pure acetone solution, and tap water) were considered for this experiment. Six replicates with 10 *T. evanescens* adult parasitoids per replicate (treated tube) were performed for each treatment. One hour later, all adult parasitoids exposed to acetone pesticide, pure acetone solution, or tap water were moved to a new clean tube with no acetone or pesticide solution, in which a honey solution was deposited on its plastic strip. Then, 6 h after parasitoid exposure to treatments, adult *T. evanescens* mortality was reported under a binocular microscope. The corresponding wasp was considered dead when no parasitoid movement was observed on the glass wall.

### 2.4. Data Analysis

Because some datasets showed complete separation or overdispersion or both in a logistic regression or model assumptions of a linear model conducted with arcsin square root transformed data were violated, we summed up the number of emerged or dead individuals per treatment within a dataset and conducted Pearson’s Chi-Square (χ^2^) test or, if expected values were below five, Fisher’s exact test without continuity correction by using the “stats” package [34]. Multiple pairwise two-sided Fisher’s exact tests without continuity correction were performed per observation time as post hoc tests and to achieve odds ratio estimates using the ”rstatix” package [35]. Adjustment of *p*-values to correct for multiple comparisons was performed using the Holm–Bonferroni method [36] available in the “stats” package. Compact letter displays [37] were generated with the cldList command of the ”rcompanion” package [38]. All statistical analyses were performed using the R software, version 4.2.2 [34], and RStudio, version 2022.12.0.353 [39].

## 3. Results

### 3.1. Effect of Pesticides on Parasitoid Emergence

**Figure 1:**
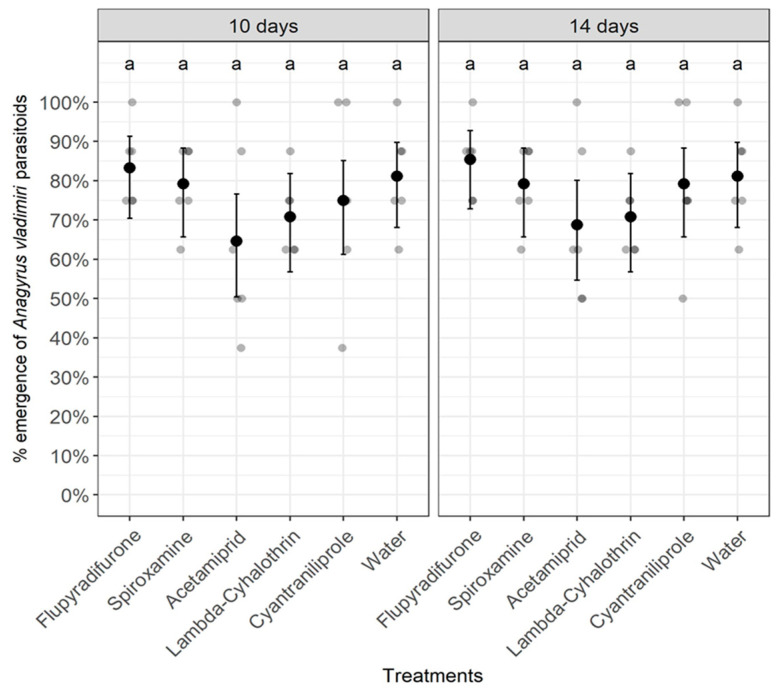
Effect of pesticides on the emergence of the parasitoid *Anagyrus vladimiri* at 10 and 14 days after treatment. Treatments with the same letter for each time point do not differ significantly (Pearson’s Chi-Square (χ^2^) test).

**Figure 2:**
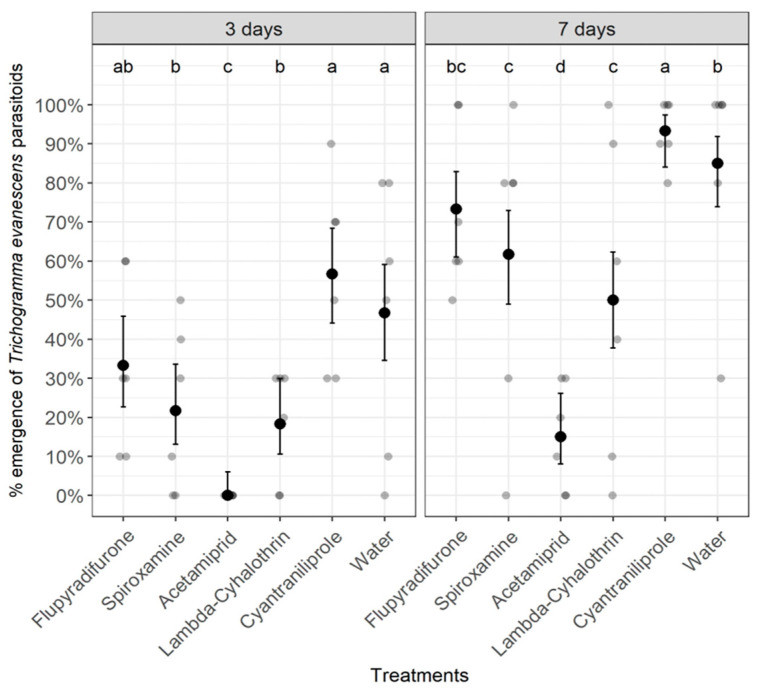
Effect of pesticides on the emergence of the parasitoid *Trichogramma evanescens* at 3 and 7 days after treatment. Treatments with the same letter for each time point do not differ significantly (Pearson’s Chi-Square (χ^2^) test).

Statistical analyses revealed that there was no significant effect of pesticides on the development of the pupal stage inside mealybug mummies or the emergence of the parasitoid *A. vladimiri* either at 10 days (Chi-Square = 6.492, df = 5, *p* = 0.2612) or 14 days after treatment (Chi-Square = 5.583, df = 5, *p* = 0.3489) (Figure 1). By contrast, all pesticides, except the diamide insecticide cyantraniliprole, significantly affected the development of the pupal stage and the emergence of the parasitoid *T. evanescens* either at 3 days (Chi-Square = 60.758, df = 5, *p* < 0.0001) or 7 days after treatment (Chi-Square = 102.654, df = 5, *p* < 0.0001) (Figure 2). Overall, the lowest percentages of *T. evanescens* emergence at both 3 and 7 days after treatment were obtained when pupae were treated with the neonicotinoid insecticide acetamiprid. However, the percentages of *T. evanescens* emergence after applying the fungicide spiroxamine or either the insecticide lambda-cyhalothrin or the insecticide flupyradifurone were significantly higher than those obtained in the acetamiprid treatment but significantly lower than those observed in the cyantraniliprole treatment (Figure 2).

### 3.2. Effect of Pesticides on the Survival of Emerged A. vladimiri Parasitoids

**Figure 3:**
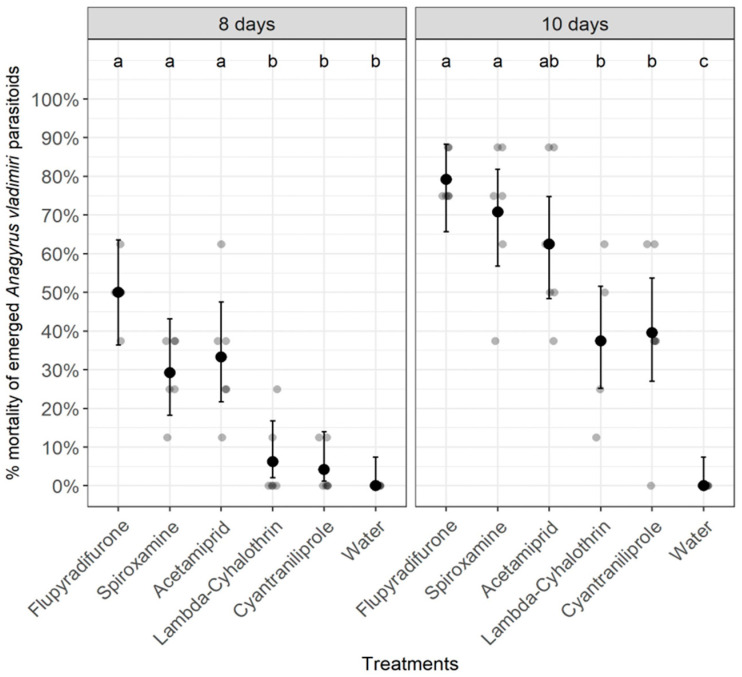
Effect of pesticides on the survival of emerged *Anagyrus vladimiri* parasitoids at 8 and 10 days after treatment. Treatments with the same letter for each time point do not differ significantly (Pearson’s Chi-Square (χ^2^) test).

There was a significant effect from treatment on the survival of emerged *A. vladimiri* parasitoids either at 8 days (Chi-Square = 58.939, df = 5, *p* < 0.0001) or 10 days after treatment (Chi-Square = 80.5, df = 5, *p* < 0.0001) (Figure 3). As shown in Figure 3, at 8 days after treatment, both insecticides flupyradifurone and acetamiprid and the fungicide spiroxamine induced the highest mortality percentages of emerged *A. vladimiri* parasitoids compared with lambda-cyhalothrin, cyantraniliprole, or the untreated control (tap water) treatments. Additionally, at 10 days after treatment, the application of either the insecticide flupyradifurone or the fungicide spiroxamine resulted in the highest mortality percentages for all emerged parasitoids compared with all other treatments (Figure 3).

### 3.3. Contact Toxicity of Pesticides toward Adult Parasitoids

**Figure 4:**
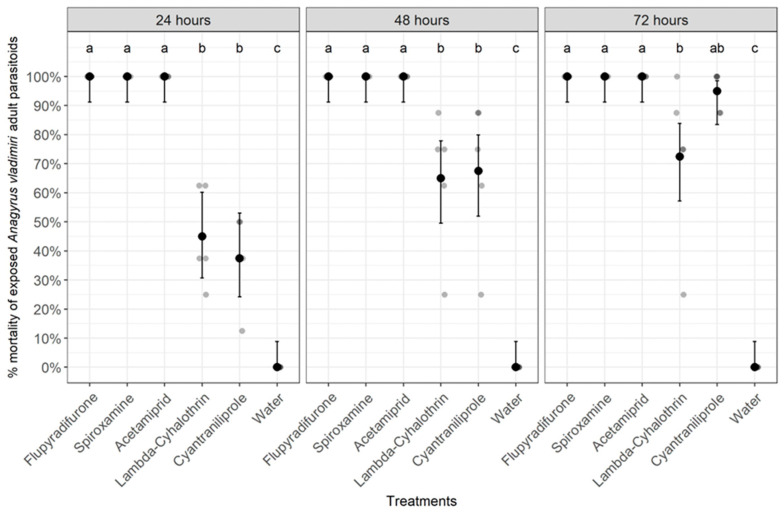
Contact toxicity of pesticides on adults of the parasitoid *Anagyrus vladimiri* at 24, 48, and 72 h after treatment. Treatments with the same letter for each time point do not differ significantly (Pearson’s Chi-Square (χ^2^) test).

All pesticides significantly affected the survival of exposed *A. vladimiri* adult parasitoids at either 24 h (Chi-Square = 156.592, df = 5, *p* < 0.0001), 48 h (Chi-Square = 151.172, df = 5, *p* < 0.0001), or 72 h after treatment (Chi-Square = 182.609, df = 5, *p* < 0.0001) (Figure 4). On all post-treatment assessment dates, the highest percentages, reaching 100%, of parasitoid mortality were obtained in the flupyradifurone, acetamiprid, and spiroxamine treatments, while the lowest mortality percentages were observed in lambda-cyhalothrin, cyantraniliprole, or untreated control treatments (Figure 4). According to the IOBC pesticide classes of toxicity based on laboratory trials, either the insecticide flupyradifurone or the insecticide acetamiprid, and the fungicide spiroxamine (all with overall mortality % equal to 100%) were classified as harmful (IOBC toxicity category 4), while both insecticides, lambda-cyhalothrin (overall % mortality equal to 60%) and cyantraniliprole (overall % mortality equal to 66%), were classified as slightly harmful (IOBC toxicity category 2) to *A. vladimiri* adults (Table 2).

**Figure 5:**
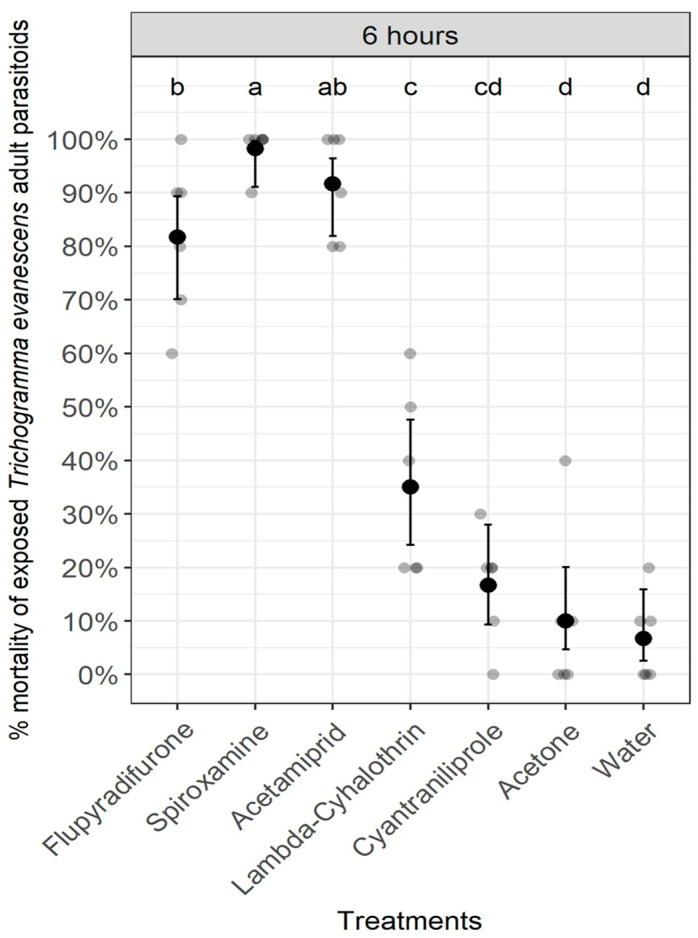
Contact toxicity of pesticides on adults of the parasitoid *Trichogramma evanescens* at 6 h after treatment. Treatments with the same letter do not differ significantly (Pearson’s Chi-Square (χ^2^) test).

Likewise, the pesticides had a significant impact on the survival of exposed *T. evanescens* adults at 6 h after treatment (Chi-Square = 237.184, df = 6, *p* < 0.0001) (Figure 5). The highest percentages of *T. evanescens* adult mortality were obtained in the flupyradifurone, acetamiprid, and spiroxamine treatments, while the fungicide spiroxamine induced significantly higher mortality percentages than flupyradifurone or acetamiprid, similar to the results obtained for contact toxicity toward *A. vladimiri* adults. On the other hand, the lowest pesticide mortality percentages for *T. evanescens* adults were observed in both the lambda-cyhalothrin and cyantraniliprole treatments, for which percentages of mortalities were significantly different compared with those observed in both the acetone and tap water treatments (Figure 5).

## 4. Discussion

Chemical pesticides that have long been used to control a wide range of crop insect pests and pathogens remain the most common pest management options worldwide [40,41,42]. This reality has long been the case in vineyards, where a plethora of pesticide active substances have been applied to cope with major grapevine insect pests, mainly including mealybugs, moths, leafhoppers, and the spotted wing drosophila [3,6,17,43]. Nevertheless, the overuse of non-selective pesticides can always exhibit detrimental side effects on non-target beneficial arthropods, which negatively impact biological control programs and vital ecosystem services [6,41,44,45]. Therefore, risk assessments of the toxicity of chemical pesticides toward natural enemies are mandatory for achieving sustainable IPM programs in vineyards.

In this context, the toxicity of five pesticides commonly applied in European vineyards toward the pupae and adults of *A. vladimiri*, a parasitoid of *P. ficus*, and *T. evanescens*, a parasitoid of *L. botrana*, was assessed in the present study. Our results demonstrated that none of the tested pesticides negatively impacted the pupal stage of *A. vladimiri* inside mealybug mummies or its emergence. Conversely, the survival of emerged *A. vladimiri* parasitoids was significantly affected by pesticides until 10 days after treatment, with either the insecticide flupyradifurone or the insecticide acetamiprid, and the fungicide spiroxamine being more toxic to emerged parasitoids compared with the insecticides lambda-cyhalothrin and cyantraniliprole. Previous studies have shown that the insecticides chlorpyrifos-methyl, Prev-Am^®^, and spirotetramat, commonly used to control *P. ficus* in Mediterranean vineyards, do not adversely impact the development of the pupal stage of the parasitoid *A. vladimiri* inside vine mealybug mummies or its emergence [6,46]. However, according to Mgocheki and Addison [47], emerged *A. vladimiri* individuals died as they gnawed an exit hole with their mandibles through the dorsal portions of vine mealybug mummies treated with fipronil or α-cypermethrin, two pesticides commonly used for ant control in South African vineyards.

Our findings, coupled with previously published research, clearly show the inability of the aforementioned pesticides to effectively reach the pupal stage of *A. vladimiri* residing in mealybug mummies, which are apparently impermeable to insecticides. This should be taken into account not only when releasing parasitoids in the field but also when these parasitoids naturally occur as pupae inside host-vine mealybugs in vineyards.

Our mortality experiments with emerged *A. vladimiri* probably show a more realistic picture of the effects that the tested pesticides could exhibit in the field compared with our experiments with adults exposed on the day of treatment. This is because it cannot be assumed that the adults hatch synchronously in the vineyard, and therefore, the adults hatched on different days are automatically confronted with pesticide residues of different ages. These experiments also reflect the different speeds of pesticide degradation and, as a consequence, the mortality in these experiments was basically lower. It is, therefore, to be expected that the two test methods might measure very different mortalities, especially for rapidly degrading pesticides. Applying insecticides when most *A. vladimiri* parasitoids are present at their pupal stage could prove to be a promising approach compared with applying insecticides in the presence of the adult stage of *A. vladimiri*. This was further confirmed by our results regarding pesticide toxicity to adult parasitoids. Indeed, we found that all pesticides significantly affected the survival of *A. vladimiri* adult parasitoids at 24 h, 48 h, or 72 h after treatment. The highest percentages of mortality, reaching 100%, were obtained in the flupyradifurone, acetamiprid, and spiroxamine treatments, which were classified as harmful pesticides, whereas the lowest mortality percentages were observed in lambda-cyhalothrin and cyantraniliprole, which are classified as slightly harmful pesticides. Hence, the application of the pesticides flupyradifurone, acetamiprid, and spiroxamine should be avoided when *A. vladimiri* is present at its adult stage in vineyards. The highest parasitoid mortality percentages (100%) obtained in our study after applying flupyradifurone, acetamiprid, or spiroxamine are comparable to the mortality percentage induced by the organophosphate insecticide chlorpyrifos-methyl toward *A. vladimiri* adults [46]. The same authors demonstrated that both insecticides Prev-Am^®^ and spirotetramat can be rated harmless (IOBC category 1) toward *A. vladimiri* adult parasitoids [46].

In contrast to what we found in the case of *A. vladimiri*, we showed that all pesticides, except the diamide insecticide cyantraniliprole, significantly affected the development of the pupal stage and the emergence of the parasitoid *T. evanescens*. More specifically, the lowest percentages of *T. evanescens* emergence at both 3 and 7 days after treatment were obtained when pupae were treated with the neonicotinoid insecticide acetamiprid compared with the pesticides flupyradifurone, lambda-cyhalothrin, and spiroxamine. Previous laboratory experiments also demonstrated that cyantraniliprole is selective (harmless) to pupae of another *Trichogramma* parasitoid, *T. atopovirilia* Oatman and Platner [48]. However, according to Saber [49], similar to our results on the higher toxicity of acetamiprid to pupae, imidacloprid, another neonicotinoid insecticide, significantly affected the emergence of *T. cacoeciae* Marchal from the pupal stage residing inside *S. cerealella* parasitized eggs. In contrast to acetamiprid and imidacloprid, the neonicotinoid insecticide thiacloprid did not affect *T. pretiosum* emergence when eggs of *S. cerealella* enclosing the pupae of the wasps were surface-treated [50]. These findings clearly indicate that the toxicity could be directly linked to the neonicotinoid active substance being assessed, as well as to the *Trichogramma* species rather than its lepidopteran host. Furthermore, similar to our findings, the pyrethroid insecticide lambda-cyhalothrin was shown to significantly affect parasitoid emergence in *T. cordubensis* (Vargas and Cabello) adults [51]. Likewise, other pyrethroid insecticides, including alpha-cypermethrin, deltamethrin, and zeta-cypermethrin, significantly reduce the percentage of emergence for the parasitoid *T. pretiosum*, which develops in *E. kuehniella* or *S. cerealella* eggs [50]. However, similar to our results for fungicide toxicity, Hassan et al. [52] found the fungicide pyrimethanil to be harmless to pupae of the parasitoid *T. cacoeciae* within the host egg. Conversely, in contrast to our results revealing the higher toxicity of the fungicide spiroxamine toward pupae, significantly affecting emergence, Vieira et al. [51] demonstrated that both the fungicides acetamide + dithiocarbamate and basic copper sulfate did not significantly affect the emergence of the parasitoid *T. cordubensis* or survival in its adult stage.

We showed that the highest percentages of *T. evanescens* adult mortality were observed in the spiroxamine fungicide treatment, followed by flupyradifurone or acetamiprid, while the lowest percentages of mortality were observed in both the lambda-cyhalothrin and cyantraniliprole treatments. Interestingly, these findings are similar to the results obtained in experiments assessing contact toxicity toward *A. vladimiri* adults for which lambda-cyhalothrin and cyantraniliprole were classified as slightly harmful pesticides compared with the three other pesticides, which were rated as harmful. Consequently, it has been suggested that pesticide treatments using the fungicide spiroxamine or either the insecticide flupyradifurone or the insecticide acetamiprid when *T. evanescens* individuals are present at their adult stage should be avoided; otherwise, the biocontrol action of these parasitoids could be significantly compromised. However, interestingly, insecticide treatments in vineyards using cyantraniliprole could be compatible with field releases of the parasitoid *T. evanescens*. Similar to our results, cyantraniliprole was also considered selective (harmless) to adults of *T. atopovirilia* [48]. As such, chlorantraniliprole, another diamide insecticide, did not significantly affect the survival of *T. pretiosum* adult parasitoids, whereas the neonicotinoid insecticide acetamiprid caused 82% mortality in *T. pretiosum* adults within 24 h of exposure to dried pesticide residues [53]. Moreover, Saber [49] showed that imidacloprid, another neonicotinoid insecticide, was harmful to adults of *T. cacoeciae* Marchal, while Tai et al. [54] found imidacloprid to be moderately harmful to *T. ostriniae* adults. As demonstrated in the present study, fungicides were already shown to exhibit high contact toxicity toward the *Trichogramma* sp. adult stage, classifying them as harmful or moderately harmful pesticides. Indeed, fungicides such as sulfur (particularly wettable sulfur), used for controlling powdery mildew, have been shown to be harmful to adults of *T. carverae* (Oatman and Pinto), an egg parasitoid of the light brown apple moth, *Epiphyas postvittana* (Walker) occurring in Australian vineyards [55]. As such, the fungicide pyrimethanil was found to be moderately harmful to the adult stage of *T. cacoeciae* [52], while the two fungicides propiconazole and difenoconazole were shown to be moderately harmful to *T. ostriniae* adults [54]. Conversely, fungicides are not always harmful to the adult stage of *Trichogramma* sp. parasitoids. For example, both the fungicides praclostrobin, a methoxy-carbamate, and trifloxystrobin + tebuconazole, a mandelamide, do not significantly affect the survival of *T. pretiosum* adults [53].

Overall, of great importance is cautiously incorporating the most selective (safer) pesticides into IPM programs against both pests *P. ficus* (involving releases of the parasitoid *A. vladimiri*) and *L. botrana* (involving releases of the parasitoid *T. evanescens*) in vineyards and to avoid using hazardous pesticides. As such, this should also be applicable to naturally occurring *A. vladimiri* and *T. evanescens* parasitoids or closely related species for which the biocontrol action should be conserved by avoiding the application of harmful pesticides and prioritizing the use of reduced-risk, safer pesticides. In both cases, the life stage of the parasitoid should be carefully considered before triggering pesticide treatments against vine mealybugs, as there are differences in toxicity between pupal and adult stages, especially in the case of *A. vladimiri*. In this context, it is worth noting that the degree of persistence of a pesticide action in the field should always be taken into account. In this context, understanding the effects of chemical pesticides over time can provide information on how to separate spray applications from releases of parasitoids [55]. Ideally, combining pesticide treatments using either insecticide lambda-cyhalothrin or cyantraniliprole with releases of *A. vladimiri* to control vine mealybugs or *T. evanescens* to control grapevine moths could be recommended, as it would effectively promote sustainable IPM programs in grape-growing areas worldwide. By contrast, applying hazardous pesticides such as the neonicotinoid insecticide acetamiprid, the butenolide insecticide flupyradifurone, or the fungicide spiroxamine should be avoided when the aforementioned parasitoids are released or occur naturally in vineyards. From this perspective, there is always a growing, urgent need to develop and implement, in combination with augmentative field releases of parasitoids, additional effective biorational control options such as prophylaxis and cultural practices, pheromone-based mass trapping or mating disruption, and applications of biopesticides [4,18,56,57,58] as sustainable alternatives to the frequent application of broad-spectrum chemical pesticides in vineyards. Such a practical scenario would further strengthen the biocontrol action of both parasitoids and enhance overall IPM programs against both insect pests in vineyards. Considering that the present study assessed lethal effects on the pupae and adults of *A. vladimiri* and *T. evanescens*, future laboratory studies will focus on elucidating the sublethal effects of all tested pesticides on both parasitoids.

## 5. Conclusions

Our results provided evidence that both the insecticides acetamiprid and flupyradifurone and the fungicide spiroxamine are harmful to adults of the parasitoids *A. vladimiri* and *T. evanescens*, whereas both the insecticides lambda-cyhalothrin and cyantraniliprole proved to be safer toward these parasitoids. Consequently, both the pyrethroid lambda-cyhalothrin and the diamide cyantraniliprole could be successfully incorporated into sustainable IPM programs against *P. ficus* and *L. botrana* populations in vineyards involving conservation or augmentative biological control using *A. vladimiri* and *T. evanescens* or closely related species.

## Figures and Tables

**Table 1:** The five pesticides tested for their toxicity: their classification, modes of action, commercial names, and target pests or pathogenic fungi (diseases) in European vineyards. Classification and modes of action are written according to the standards of the Insecticide Resistance Action Committee [28] and the Fungicide Resistance Action Committee [29].

Pesticide Active Ingredient (a.i.) or Common Name	Main Group/Primary Site or Mode of Action	Sub-Group, Class, Chemical, or Biological Group	Trade Name (Manufacturer)	Target Pest(s) or Pathogenic Fungi (Diseases) in Vineyards
Cyantraniliprole	Ryanodine receptor modulators/nerve and muscle action	Diamides	Exirel^®^ (FMC, Germany)	Spotted wing drosophila
Flupyradifurone	Nicotinic acetylcholine receptor (nAChR) competitive modulators/Nerve action	Butenolides	Sivanto^®^ Prime (Bayer Crop Science, Germany)	Mealybugs and leafhoppers
Lambda-Cyhalothrin	Sodium channel modulators/nerve action	Pyrethroids	Karate Zeon^®^ (Syngenta, Germany)	Spotted wing drosophila, grapevine moths, and leafhoppers
Acetamiprid	Nicotinic acetylcholine receptor (nAChR) competitive modulators/nerve action	Neonicotinoids	Mospilan^®^ (FMC, Germany)	Spotted wing drosophila
Spiroxamine	Amines (“morpholines”) (SBI: Class II)/sterol biosynthesis in membranes	Spiroketal-amines	Prosper^®^ (Bayer Crop Science, Germany)	Powdery mildew

**Table 2:** Contact toxicity of pesticides on adults of the parasitoid *Anagyrus vladimiri*, expressed by mortality % after treatment. Pesticides are classified according to the IOBC classes of toxicity (class 1: harmless (<30%); class 2: slightly harmful (30–79%); class 3: moderately harmful (80–99%); class 4: harmful (>99%).

Treatment	Overall Mortality % after Treatment	IOBC Class of Toxicity
Flupyradifurone	100 a	4
Acetamiprid	100 a	4
Spiroxamine	100 a	4
Cyantraniliprole	66 b	2
Lambda-cyhalothrin	60 b	2
Control (water)	0 c	-

Means followed by the same letter do not differ significantly (Pearson’s Chi-Square (χ^2^) test).

## Data Availability

The datasets generated during and/or analyzed during the current study are available from the corresponding authors upon reasonable request.
